# A Retrospective Analysis of Malaria Trends in Leka Dulecha Health Center over the Last Ten Years (2013-2022), Western Oromia, East Wollega Zone

**DOI:** 10.1155/2023/6635249

**Published:** 2023-08-07

**Authors:** Temesgen Tafesse, Chimdessa Tolera, Desalegn Amenu

**Affiliations:** ^1^Microbiology and Microbial Biotechnology, Armauer Hansen Research Institute, Addis Ababa, Ethiopia; ^2^East Wollega Zonal Health Center, Leka Dulecha Health Center, Nekemte, Ethiopia; ^3^Jimma University, College of Natural Science, Biology Department, Microbiology (Food Microbiology), Ethiopia; ^4^Wollega University, Ethiopia

## Abstract

**Background:**

Malaria is a serious public health concern in the world, and it causes a major socioeconomic problem in Ethiopia. Malaria data trend analysis of health facilities is useful to understand the prevalence and incidence of malaria cases and implementing evidence-based malaria control strategies. Hence, the main objective of this study was to investigate the malaria trends over the last ten years (2013-2022) at Leka Dulecha Health Center, East Wollega Zone, Western Oromia. *Methodology*. A retrospective study was conducted at Leka Dulecha Health Center to determine the trends of malaria prevalence by considering the malaria registration laboratory logbook for the last ten years from 2013 to 2022. Hence, to do this, sociodemographic data, years, months, and malaria prevalence were collected using a predesigned data collection sheet recorded from perspective between years.

**Results:**

In the last ten years, a total of 30,576.00 suspected malaria cases were examined at Leka Dulecha Health Center, and out of these, 7,413.00 (24.24%) confirmed malaria cases were reported. In this health center, malaria cases were reported among both sexes and all age categories, but male (3,951.00, 54%) and age groups ≥ 15 years (3,994, 54%) were the most affected. The highest peak of malaria cases was reported during the autumn season (September, October, and November) followed by the spring season (March, April, and May) in the years of 2013 and 2007. In this study, the prevalence of malaria species was identified as Plasmodium falciparum, Plasmodium vivax, and mixed cases, with 5,014 (68%), 1,123 (15%), and 1,848 (25%), while Plasmodium falciparum was reported as the highest recorded cases.

**Conclusion:**

Males and above 15 years old were more affected than the others. The highest peak malaria prevalence appeared from September to December of 2017 and 2013 years. Therefore, proper planning, implementation, and monitor of malaria prevention and control activities should be strengthened at all levels.

## 1. Introduction

Despite the fact that malaria is a treatable disease, it has remained a major public health challenge that affects the lives of billions of people worldwide [1]. In 2020, the WHO reported that there were approximately 241 million cases of malaria and 627, 000 malaria-related deaths. This indicates that malaria cases and deaths in 2020 were increased by 14 million and 69,000, respectively, compared to the 2019 report. Sub-Saharan Africa bores the largest share of the global malaria burden, accounting for an estimated 95% of malaria cases and 96% of malaria-related deaths in 2020 [2].

More than 68% of Ethiopia's landmass is suitable for malaria transmission, and as a result, more than 60 million of the people live in malaria-prone areas [3]. Malaria risk and transmission intensity are significantly affected by seasonal variation with latitude, longitude, and ecological difference [4]. Malaria transmission peaks in most Ethiopian regions from September to December following a heavy summer rainy season, with lower transmission lasting from April to May following a short rainy season [5]. Furthermore, the prevalence and incidence of malaria vary according to sociodemographic risk factors such as age and gender [6]. The country is prone to cyclic epidemics every 5 to 8 years due to unstable transmission patterns and environmental changes [1, 5–7].

Despite the general public health significance and widespread incidence of malaria in several parts of Wollega Zone, the general trend of malaria prevalence in Leka Dulecha Health Center in particular has not been thoroughly studied. Analyzing the morbidity pattern of malaria in malaria endemic areas would aid in understanding the dynamics of disease transmission and evaluating the efficacy of malaria intervention options to reduce disease burden in a locality. As a result, the purpose of this study was to assess the trends of malaria and transmission patterns over sex, age, and season at Leka Dulecha Health Center in East Wollega Zone over the last ten years.

## 2. Materials and Methods

### 2.1. Study Area and Period

**Figure 1:**
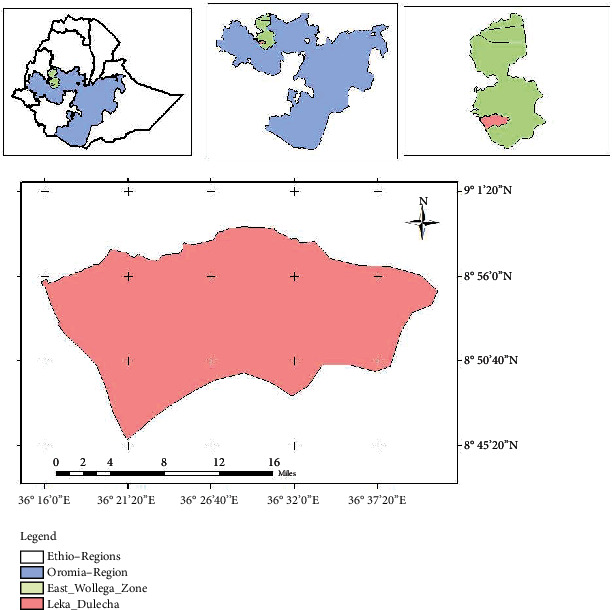
Map of the study area, East Wollega Zone, Leka Dulecha District, 2022.

The study was conducted in East Wollega Zone, Leka Dulecha District, at public health facilities of Leka Dulecha Woreda. There are 21 rural and 2 urban administrative kebeles. The total population of woreda based on the 2014 population projection is 105,412 of which 53,128 are females and 52,284 are males. There are four health centers and 22 rural and 2 urban health posts with a total of 169 healthcare providers and 31 support staff. The woreda public health facility has responsibility for providing a comprehensive package of preventive, promotive, curative, and rehabilitative health services to the community at large (Figure 1).

### 2.2. Study Design

A cross-sectional public health facility study was conducted at the Leka Dulecha Health Centre, Leka Dulecha District, East Wollega Zone, Oromia Regional State, from June to September 2022.

### 2.3. Source of Information

The malaria prevalence rate was calculated using data from a malaria laboratory registered logbook available at a health facility. This data was used as a source of information in the study to provide malaria diagnosis and treatment. The variables captured are the same because the center used a similar database for all patients.

### 2.4. Malaria Trend Analysis

Data from the registered book were transferred to establish checklists to determine trends in malaria prevalence at the health center. The date of diagnosis, patient address, gender, age, and diagnostic tools, as well as the diagnosis result and parasitic species, were all variables in the study.

### 2.5. Data Quality Control

To maintain data quality, data collectors and supervisors were trained for two days prior to the study. They were also taught about the study's data collection tools (variables of interest, rational, objective, and significance), and the principal investigators monitored the entire process daily, including data collection and entry, to ensure accuracy and consistency.

### 2.6. Data Collection and Data Analysis

Using a data collection sheet, malaria data for ten years were extracted from a laboratory logbook, including year, month, sex, age, residence, total number of BFS examined, and species type (P. falciparum, P. vivax, and mixed infections). The data was then analyzed using SPSS version 26 software. The association of Plasmodium species with age group, sex, and residence was determined using mean analysis. The overall trend of malaria prevalence and malaria species distribution with residence and season was depicted using graphs.

## 3. Results

### 3.1. Annual Trends of Malaria Burden

In the past 10 years (2013–2022), Leka Dulecha Health Center has diagnosed 30,576.00 people with malaria suspicion (Table 1). Of these, 7,413 (24.24%) had microscopically proven malaria cases. An average of 742 confirmed cases of malaria was reported at this health center each year. The number of malaria suspected cases progressively decreased from 2015 to 2018, and then, it sharply increased during 2019-2022 (Table 1). The highest prevalence of malaria cases was observed in the year 2021 in which 1530 patients were recorded and the lowest prevalence was observed during 2017 and 2019, with 311 and 318, respectively.

In all cases, over the last ten years, *Plasmodium falciparum* was the leading malaria parasite identified with an estimated prevalence of 16.70% followed by *Plasmodium falciparum-Plasmodium vivax* mixed infection, 6.04%. The remaining 3.67% cases of malaria were due to *Plasmodium vivax* infection (Table 1).

### 3.2. Trends of Malaria Cases in Leka Dulecha Health Center (2013 to 2022)

**Figure 2:**
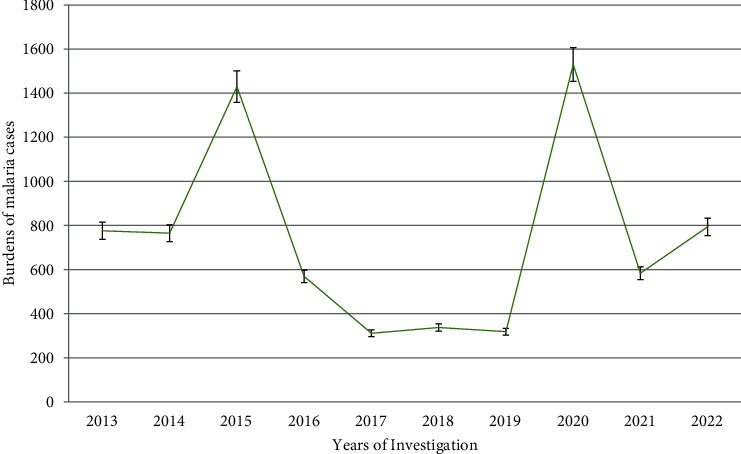
Trends of malaria prevalence in Leka Dulecha Health Center, East Wollega Zone (2013-2022).

In the past ten years (2013-2022), the prevalence of malaria cases showed great variation and the trend of malaria was highly fluctuating across years ranging from 52.00% to 9.40%, in 2021 and 2017, respectively. Hence, the highest peak of malaria cases was reported in 2021 (52.00%), followed by 2020 (38.43%) and 2013 (38.09%), respectively. Although the trend of malaria prevalence was observed with an insubstantial and inconsistent distribution across years, a generally steady declining trend of malaria prevalence was observed from 2015 to 2017 with an estimated prevalence of 38.43%, 21.50%, and 9.40%, respectively. But, during the years, 2013 and 2015, constant malaria cases were documented, while in 2018 about 38% prevalance rate was recorded. On the other hand, a significant increment trend of malaria prevalence was observed from 2017 (9.40%) to 2020 (52.00%) (Figure 2).

### 3.3. Seasonal Distribution of Malaria at Leka Dulecha Health Center

The seasonal distribution of malaria at Leka Dulecha Health Center was assessed according to the malaria cases reported in all twelve months and four seasons throughout the ten years. In line with seasons, the highest cases were reported during summer (June, July, and August) (32.20%) and the lowest during spring (March, April, and May) (20.70%), respectively. Besides, the months November, July, and June had the highest malaria cases, with 17.70%, 16.40%, and 12.45%, respectively, and September and March had the lowest malaria cases, 5% and 3.90%, respectively (Table 2).

### 3.4. Distribution of Malaria in relation to Sex and Age at Leka Dulecha Health Center

In this study, the prevalence of malaria over the past ten years showed great variation among different age groups. Hence, from the total ages, those with age greater than 15 years old were the more affected groups followed by age between 6 and 15 groups. Therefore, regarding the trend analysis of malaria distribution among age groups, individuals who were above 15 years old were identified as the most affected population across all years (2013-2022) (Table 3).

**Figure 3:**
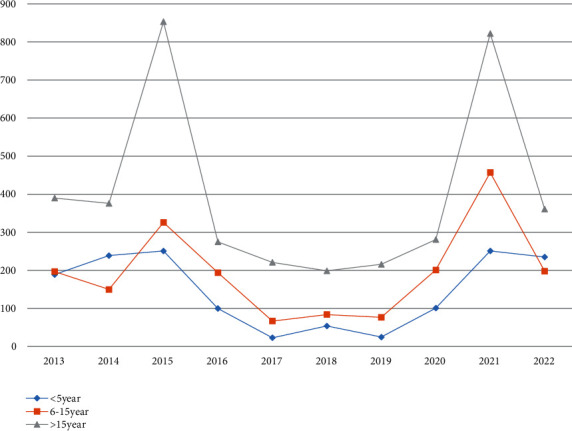
Distribution of malaria in different age groups at Leka Dulecha District, 2013-2022.

Along with the age groups, this finding showed that males were identified as the most affected sex category which was more affected by malaria cases with the estimated prevalence rate of 53.34% (*n* = 3948/7413); females and males shared the highest portion of malaria cases throughout all past ten years (2013-2022) (Figure 3).

## 4. Discussion

Over the last past ten years, totally 30,576 malaria suspected people were diagnosed using blood film examination at Leka Dulecha Health Center from 2013 to 2022. Out of these, 7,413 (24.24%) of malaria cases were reported; hence, 24.24% of malaria confirmed cases was observed. So, in this study, the overall prevalence of malaria cases at Leka Dulecha Health Center was 24.24%. On the other hand, the maximum and minimum prevalence rate of malaria was observed in 2015 and 2017, 1430 (38.43%) and 311 (9.40%), respectively. This study is higher than research reported by Tilahun, Macedo, Belew, Desalegn, Getachew, and Lema with the overall prevalence of malaria infection 11.54%, 15.57%, 16.34%, 16.60%, 17.00%, and 23.20%, respectively. In contrast to these results, this finding is lower than the study conducted by [8–10] with the prevalence rate of 33.8%, 39.60%, and 53.68%, respectively. In general, this disparity could be attributed to differences in sample size, altitude, climate, malaria treatment availability, and utilization practices. Over the last ten years, 2013-2022, the trends of malaria prevalence did not show proportional trends and fluctuate over the ten years, but the ultimate decline rates of malaria cases were observed in the years 2015-2017. On the other hand, the highest peak of cases was reported in 2020. In this year, the highest cases were more related to a lack of adequate treatment and an overload of COVID-19 cases in the district over the last three years.

Concerning to the prevalence of malaria species, *Plasmodium falciparum* was detected as the predominant (68%), followed by *Plasmodium vivax* (21%), and 11% accounted for mixed cases. This result is comparable to the study conducted at Motta Health Center, where *Plasmodium falciparum* was reported as the predominant malaria parasite (60.9%), followed by *Plasmodium vivax* which accounted 31.1% prevalence rate. In addition, with supporting the present finding [11], reported that *Plasmodium falciparum* was the most predominant parasite detected at Metema Hospital with the prevalence rate of 90.7%, followed by *Plasmodium vivax* (9.00), respectively. This finding is more similar to national malaria reports highlighted in Ethiopia, which reports the dominance of *Plasmodium falciparum* [12].

In contrary to these findings, a retrospective study conducted in Arsi Negele revealed that *Plasmodium vivax* was the highest prevalent parasite (74%), followed by *Plasmodium falciparum* with the prevalence rate of 19.8%, respectively [13]. In addition, the study conducted in Diba Health Center also showed that *Plasmodium vivax* was still the dominant parasite which accounted 75% followed by *Plasmodium falciparum* (25%) [8]. Besides, the study conducted in Butajira Health Center revealed that *P. vivax* was the most dominant parasite reported from the study area, with the prevalence rate of 86.5% followed by *Plasmodium falciparum* (12.4%) [14].

This shows that, in all cases of the study sites, *Plasmodium vivax* was reported as the most predominant species than *Plasmodium falciparum*. This study is contemporaneous with the rate of malaria distribution in Ethiopia, in which both *Plasmodium falciparum* and *Plasmodium vivax* were the two most distributed malaria parasites with 60% and 40% of prevalence, while *Plasmodium falciparum* share the highest portion [15].

Regarding the malaria distribution with age groups, malaria cases were reported among all age groups. However, the rate of prevalence was very high among age category > 15 years old followed by 6-15 years old and children under the age of five. In line with this study, Meku reported the same result with this findings in which a high prevalence of malaria cases also was found among age greater more than 15 years old. This may be resulted due to improper usage of bed net or daily activities like planting and harvesting that may increase malaria incidence among working age group and working adults [10].

Besides, males were found to be the most profound group than females to contract malaria in this retrospective analysis, which corresponded to the gender distribution of malaria cases. This finding is also more similar with the research reported by [7, 16–18]; in all cases, the authors reported that males were more likely to be contradicted with malaria cases than females. So, this reason may be related to the fact that males are more engaged in activities outside their residence area, migration which make them more prone to infective mosquito bites compared to female counter parts which are mostly at home and are not exposed to malaria areas and protected from such infective bites [2].

In the study area, the prevalence of malaria cases was observed in each season of each year, 2013-2022. The highest prevalence of malaria cases was observed during the summer season (June, July, and August) with 32.20% prevalence rate, while the lowest prevalence was recorded during the spring season (September, October, and November) with 17.70% prevalence rate. This study is contradicted with other studies done in Assosa Hospital, Metema Hospital, and Kola Diba Health Center in which the highest peak of malaria transmission was reported in spring followed by summer season [8, 11, 19], but it was concurrent with the study conducted by Lemma, who reported that the highest prevalence of malaria was observed during June to August [2]. Over the last ten years, the malaria trend has revealed no consistent pattern of cases in different years. Interannual and intra-annual variations in malaria transmission patterns have been observed, as there have been inconsistencies in disease burden distribution across age groups and gender. Understanding disease distribution in time and space is critical for effective interventional planning.

## Figures and Tables

**Table 1:** Microscopically confirmed malaria cases at Leka Dulecha Health Center from 2013 to 202 (*n* = 30,576.00).

Years	Total blood examined	Confirmed cases, *n* (%)	*Plasmodium falciparum*	*Plasmodium vivax*	Mixed
2013	2142	776 (38.09%)	487 (22.80%)	182 (8.50%)	10 (5.00%)
2014	2037	765 (21.10%)	564 (28.00%)	133 (6.5%)	68 (3.50%)
2015	3624	1430 (38.43%)	1013 (28.00%)	296 (8.20%)	121 (3.40%)
2016	3721	569 (21.50%)	357 (10.00%)	121 (3.30%)	91 (2.50%)
2017	2678	311 (9.4%)	186 (7.00%)	68 (2.60%)	57 (2.20%)
2018	3332	337 (12.54%)	199 (6.00%)	91 (2.80%)	47 (1.40%)
2019	2689	318 (10.00%)	188 (7.00%)	71 (2.80%)	59 (2.20%)
2020	3188	583 (14.00%)	364 (11.50%)	131 (4.20%)	92 (2.80%)
2021	4217	1530 (52.00%)	1087 (26.00%)	313 (7.50%)	130 (3.08%)
2022	2948	794 (27.00%)	569 (19.40%)	144 (5.00%)	81 (2.80%)
Total		7,413.00 (24.24%)	5,014 (68%)	1,550 (21%)	850 (11%)

**Table 2:** Seasonal distribution of malaria cases at Leka Dulecha Health Center, 2013-2022.

S/N	Months	Seasons	Total positive	P. falciparum	P. vivax	Mixed
(1)	September	Autumn	370	272	69	29
(2)	October		707	468	156	83
(3)	November		1310	922	268	170
(4)	December	Winter	729	454	184	91
(5)	January		524	345	102	77
(6)	February		393	275	83	35
(7)	March	Spring	290	199	55	36
(8)	April		580	370	127	83
(9)	May		658	387	163	108
(10)	June	Summer	923	614	202	107
(11)	July		1211	844	260	107
(12)	August		687	475	128	83

**Table 3:** Distribution of malaria at Leka Dulecha Health Center in relation to sex and age, 2013 to 2022.

Parasites	Sex	Age			Total
		<5 years	5-15 years	>15 years	
P. falciparum	Male	510.00	648.00	1,420.00	2,578.00
	Female	487.00	630.00	1,319.00	2,436.00
P. vivax	Male	140.00	270.00	490.00	900.00
	Female	160.00	183.00	306.00	649.00
Mixed	Male	110.00	100.00	260.00	470.00
	Female	61.00	120.00	199.00	380.00
Total		1,468.00	1,951.00	3,994.00	7,413.00

## Data Availability

All data used in this study were included in the manuscript, table, and figures; there are no any data or supplementary data.
